# Allicin attenuates myocardial apoptosis, inflammation and mitochondrial injury during hypoxia-reoxygenation: an in vitro study

**DOI:** 10.1186/s12872-021-01918-6

**Published:** 2021-04-21

**Authors:** Xinyi Deng, Peng Yang, Tong Gao, Mengru Liu, Xianlun Li

**Affiliations:** 1 Peking University China-Japan Friendship School of Clinical Medicine, 2 East Yinghuayuan Street, Hepingli, Beijing, 100029 China; 2 Graduate School, Peking Union Medical College and Chinese Academy of Medical Sciences, Beijing, 100730 China; 3 Department of Integrative Medicine Cardiology, China-Japan Friendship Hospital, Beijing, China

**Keywords:** Myocardial ischemia–reperfusion, Allicin, Cardiomyocytes, Apoptosis, Inflammation, Mitochondrial injury

## Abstract

**Background:**

Myocardial ischemia–reperfusion (IR) injury is a damage due to an initial reduction in blood flow to the heart, preventing it from receiving enough oxygen, and subsequent restoration of blood flow through the opening of an occluded coronary artery producing paradoxical harmful effects. The finding of new therapies to prevent IR is of utmost importance. Allicin is a compound isolated from garlic having the ability to prevent and cure different diseases, and a protective effect on the myocardium was also demonstrated. Therefore, the aim of this study was to evaluate the in vitro protective effect of Allicin against myocardial IR injury on cardiomyocytes.

**Methods:**

We established an in vitro hypoxia-reoxygenation (HR) model of primary porcine cardiomyocytes to simulate myocardial IR injury. Primary porcine cardiomyocytes were extracted from Mini-musk swines (1 day old). After a period of adaptation of at least 2–3 days, cardiomyocytes in good condition were selected and randomly divided into control group (normal oxygen for 5 h), HR group (2 h of hypoxia/3 h of reoxygenation), and HR + Allicin group (hypoxia/reoxygenation + Allicin treatment).

**Results:**

After the induction of hypoxia/reoxygenation, Allicin treatment enhanced the cell viability. Moreover, Allicin treatment resulted in a reduction of apoptosis from 13.5 ± 1.2% to 6.11 ± 0.15% compared with the HR group (p < 0.05), and the apoptosis related proteins were regulated as well, with a decreased expression of Bax, cleaved caspase-3 and cytosolic cytochrome C and an increase in Bcl-2 expression in the HR + Allicin group (all p < 0.01). Pro-inflammatory cytokines, such as interleukin-6 and tumor necrosis factor alpha were down-regulated by the treatment with Allicin (both p < 0.01). In addition, it significantly decreased intracellular reactive oxygen species generation (p < 0.01) and reduced the loss of mitochondrial membrane potential (p < 0.01). Furthermore, the expression of PPARγ coactivator-1α and endothelial nitric oxide synthase was up-regulated (both p < 0.01), while the expression of Endothelin-1, hypoxia inducing factor-1α and transforming growth factor beta was down-regulated (all p < 0.01) by Allicin treatment.

**Conclusions:**

These results suggested that Allicin protected the cardiomyocytes against HR damage by reducing apoptosis, inflammation and mitochondrial injury, thus providing a basis for its potential use in the treatment of myocardial IR.

**Supplementary Information:**

The online version contains supplementary material available at 10.1186/s12872-021-01918-6.

## Background

Myocardial infarction is the main reason for death and disability worldwide. It is due to an initial reduction in blood flow to the heart, preventing it from receiving enough oxygen. Thus, the most effective approach to treat myocardial infarction is the rapid restoration of blood flow through the occluded coronary artery via primary percutaneous coronary intervention [1]. However, the restoration of the blood flow may aggravate cardiac dysfunction due to the production of reactive oxygen species and the accumulation of proinflammatory immunocytes in the ischemic tissues, thus inducing extra damage to cardiomyocytes. Therefore, this damage is called myocardial ischemia–reperfusion (IR) injury. IR injury accounts for 50% of the final size of a myocardial infarct. Numerous studies showed that the mechanism of IR includes intracellular calcium overload, apoptosis, and inflammation [2]. Currently, there is no effective treatment for IR [3], hence, it is necessary to explore new strategies to protect the heart against IR injuries.

Allicin is a chemical compound (diallyl disulfide) extracted from garlic. Many studies demonstrated that Allicin exerts a cardioprotective effect by reducing total cholesterol, lowering blood pressure and inhibiting platelet aggregation [4, 5]. Our previous studies revealed that Allicin improves hemodynamics, reduce myocardial no-reflow area, and inhibits the expression of CD29 and CD31 in a catheter-based porcine model [6–8]. Although substantial evidence is available supporting the association between Allicin and heart protection, the exact mechanism used by Allicin to prevent cardiovascular disease remains unknown. Therefore, the objective of our study was to explore the mechanism exerted by Allicin to protect from Hypoxia/reoxygenation (HR) in an in vitro study, to provide a basis for a potential use in the treatment of myocardial IR.

## Methods

### Reagents and animals

Mini-musk swines (1 day old) were purchased from the Center of Experimental Animal of China Agricultural University. All animals used in this study were handled and treated according to the “Guide for the Care and Use of Laboratory Animals” published by the United States National Institute of Health (Publication No. 85-23, revised in 1996), and all the performed experiments were approved by the Animal Care and Use Committee of China-Japan Friendship Hospital, Beijing, China.

Allicin was purchased from Targetmol (purity 80%-95%) and the dilution was freshly prepared in sterile saline or cell growth medium as needed before each experiment. The antibodies used for western blot were the following:Bcl-2 Associated X Apoptosis Regulator (Bax) Rabbit polyclonal antibody (OM241088, 1:1000) from Omnimabs; B-cell lymphoma 2 (Bcl-2) rabbit polyclonal Antibody (BS-4563R, 1:1000) from Bioss; Cleaved caspase-3 rabbit polyclonal antibody (9661 T, 1:2000) from Cell Signaling Technology; Cytochrome C mouse monoclonal antibody (NB100-56503SS, 1:2000) from Novus; GAPDH rabbit polyclonal antibody (10494-1-AP, 1:5000) from Proteintech; HRP-conjugated mouse anti-rabbit IgG (#5127, 1:2000, Cell Signaling Technology) and HRP-conjugated horse anti-mouse IgG (#7056, 1:1000 from Cell Signaling Technology).

### Isolation of neonatal porcine cardiomyocytes

Primary cultures of neonatal porcine cardiomyocytes were obtained from the ventricles of 1 day old Mini-musk swines. After disinfection of the whole body with 0.1% bromogeramine, swines were killed by a lethal injection of pentobarbital sodium followed by exsanguination. The heart was excised under aseptic conditions, the attached tissues were removed and the residual blood was washed away with PBS. The ventricles were minced into small pieces of approximately 1–3 mm^3^, dissociated by 0.125% trypsin (Gibco, 25200072) for 5 times at 37 °C, filtered and centrifuged for 10 min at 1200 rpm at 4 °C. The obtained primary cardiomyocytes were cultured in Dulbecco’s modified Eagle’s medium (DMEM, Hyclone, SH30243.01B) supplemented with 10% fetal bovine serum (Hyclone, SH30084.03). The resuspended cells were adjusted to a concentration of 5 × 105/ml, lated in a cell culture flask and incubated at 37 °C under 5% CO2 for 1.5 h.

### Cardiomyocytes culture and grouping

**Fig. 1:**
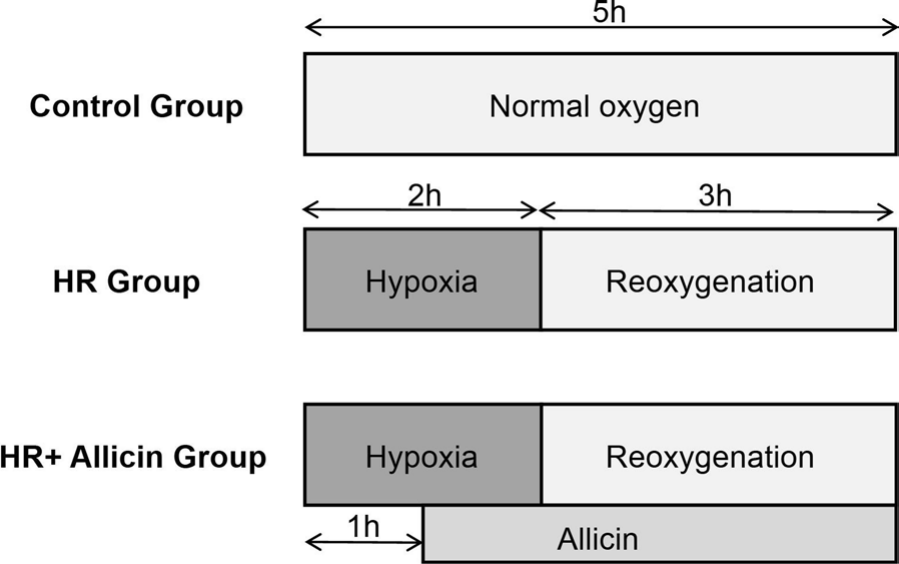
Experimental design. The cardiomyocytes in the control group were cultured under normal oxygen for 5 h. The cardiomyocytes in the HR group were cultured under hypoxic environment for 2 h, then, the normal oxygen condition was re-established and kept for 3 h. The cardiomyocytes in the HR + Allicin group were cultured under hypoxic environment for 2 h, then, the normal oxygen condition was re-established and kept for 3 h, and Allicin was added to the culture medium at the end of the first hour of hypoxia

The cardiomyocytes were cultured in 90% DMEM + 10% FBS for at least 2–3 days. Next, the ones in good condition (such as self-regulatory pulsations of 90–120 times/min) were selected and randomly divided into the following groups (n = 3): (1) control group represented by cardiomyocytes cultured under normal oxygen for 5 h; (2) HR group represented by cardiomyocytes cultured under 94% N_2_ + 1% O_2_ + 5% CO_2_ for 2 h to induce the hypoxia, and then, the normal oxygen condition was re-established in the culture and kept for 3 h to induce the reoxygenation condition; (3) HR + Allicin group represented by cardiomyocytes cultured under 94% N_2_ + 1% O_2_ + 5% CO_2_ for 2 h, then 10 μL Allicin dilution was added at the end of the first hour, and then, the normal oxygen condition was re-established in the culture and kept for 3 h (Fig. 1). At the end of the experiment, several parameters were measured as explained in the following paragraphs. The experiment was performed in triplicate and repeated three times.

### Cell viability

Cell viability was determined using the 3-(4,5-dimethylthiazol-2-yl)-2,5-diphenyltetrazolium bromide (MTT) assay (Solarbio, Beijing, China) on the cardiomyocytes of each group. Cardiomyocytes were plated into 96-well plates at a density of 1.0 × 10^4^ cells/well and treated according to the experimental design and Allicin was diluted to 5 μg/mL, 10 μg/mL, 20 μg/mL and 40 μg/mL. After removing the medium, 100 µL of MTT (0.5 mg/mL) was added to the culture and incubation continued for an additional 4 h at 37 °C. Then, the culture medium was replaced with 150 µL of dimethyl sulfoxide and the absorbance was measured at 490 nm. The cell viability of cardiomyocytes was expressed as a percentage of the control group.

### Apoptosis assay by flow cytometry

Cardiomyocytes were plated into 24-well plates at a density of 6.0 × 10^4^ cells/well and treated according to the experimental design. The apoptosis of cardiomyocytes was analyzed by flow cytometry using Annexin V/PI double staining kit (Dojindo) strictly according to the manufacturer's introduction. Cell apoptosis was measured by flow cytometry (Beckman, CytExpert).

### Western blot

Cardiomyocytes were plated into 24-well plates at a density of 6.0 × 10^4^ cells/well and treated according to the experimental design. Mitochondrial and cytosolic cytochrome C were separated using a cytochrome C releasing apoptosis assay kit (Biovision, Milpitas, CA, USA). At the end of the experiment, the cells were homogenized in IP lysis buffer (Beyotime) at 4 ℃ for 30 min. After centrifugation at 12,000 rpm for 10 min, the supernatant was transferred to a 1.5 mL centrifuge tube. Protein concentration was quantified using the BCA protein assay kit. Proteins were separated by electrophoresis on a sodium dodecyl sulfate–polyacrylamide gel and then transferred onto PVDF membrane. The membrane was blocked with 5% (w/v) nonfat dry milk in Tris-buffered saline for 2 h at room temperature, followed by incubation with the primary antibodies at 4 °C overnight. Next, the membrane was washed 3 times with 0.05% TBST and incubated with the secondary antibodies. Bands were detected using an ECL detection kit (Beyotime) following the manufacturer’s protocols. All western blots were revealed using the ChemiDoc XRS + from Bio-Rad and quantified using the IPP software (Image-Pro Plus 6.0). GAPDH was used as the loading control.

### Enzyme-linked immunosorbent assay (ELISA)

Cardiomyocytes were plated into 24-well plates at a density of 6.0 × 10^4^ cells/well and treated according to the experimental design. Interleukin-6 (IL-6), tumor necrosis factor-α (TNF-α), transforming growth factor-β (TGF-β) and endothelial nitric oxide synthase (eNOS) expression in the myocardial cells of each group was measured using the correspondent ELISA kits (Wuhan ColorfulGene Biological Technology, JYM0023Po) according to the correspondent manufacturer’s instructions.

### Reactive oxygen species (ROS) assay

Cardiomyocytes were plated into 24-well plates at a density of 6.0 × 10^4^ cells/well and treated according to the experimental design. To determine ROS level, cardiomyocytes were stained with 2′,7′–dichlorofluorescein diacetate (DCFDA) followed by washing in PBS 3 times. ROS were measured by a microplate reader (MULTISKAN MK3, Thermo) at an excitation wavelength of 488 nm, and emission wavelength of 525 nm.

### Mitochondrial membrane potential (MMP)

Cardiomyocytes were plated into 24-well plates at a density of 6.0 × 10^4^ cells/well and treated according to the experimental design. The change in MMP was evaluated by the cationic dye JC-1 (Solarbio, M8650). JC-1 forms aggregates in the mitochondria in response of the high MMP and emits a red fluorescence. When the MMP is low, JC-1 accumulates in the cytosol as a monomer, emitting a green fluorescence. Cardiomyocytes were treated with JC-1, incubated at 37 °C for 20 min and then washed. The relative ratio of red and green fluorescence was measured using a dual-wavelength microplate analyzer.

### Quantitative real-time PCR (qRT-PCR)

Cardiomyocytes were plated into 24-well plates at a density of 6.0 × 10^4^ cells/well and treated according to the experimental design. Cell total RNA was isolated using TRIzol reagent (Invitrogen) and reverse transcribed using a PrimeScript™ RT Reagent Kit (Fermentas) according to the manufacturer’s instructions. qRT-PCR was performed using an SYBR Green qPCR Reagent Kit (Thermo) according to the manufacturer’s protocol. qRT-PCR conditions were the following: 40 cycles of denaturation at 95 °C for 10 s, extension at 60 °C for 15 s and final extension at 72 °C for 20 s. Real-time fluorescence quantitative PCR instrument (V7, ABI Company) was used to perform qRT-PCR. All primers were designed using Oligo 6 primer analysis software. The primer sequences are shown in Table 1. The relative expression of the selected genes was calculated using the 2^−ΔΔct^ method. GAPDH was used as the internal control.

**Table 1:** Primer sequences used for qRT-PCR

Gene	Forward primer	Reverse primer
GAPDH	ACCTCCACTACATGGTCTACA	ATGACAAGCTTCCCGTTCTC
PGC1-α	GATGGAGACAGCTATGGTTTCA	AGTACAGCTCGAAGTCAGTTTC
ET-1	TGGAGAAACGCTGGGATAAC	TGGCCTCCAACCTTCTTATTT
HIF-1α	CCAGTCTCAGTGTGGGTATAAG	CAGACTGTGACGACTGAGAAA

### Statistical analysis

Statistical analysis was performed using SPSS 24.0 statistical software, and all data were expressed as mean ± standard deviation (± SD). The Tukey method in one-way ANOVA was used for pairwise comparison among different groups. A *p *value < 0.05 was considered statistically significant.

## Results

### Allicin enhanced cell viability

**Fig. 2:**
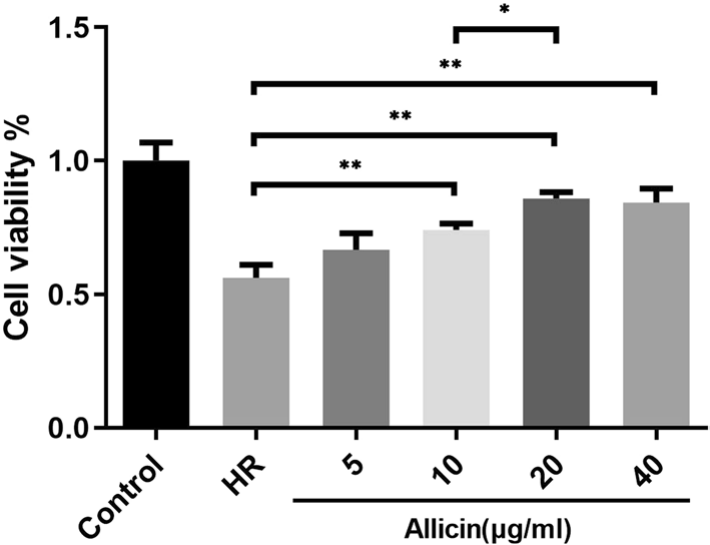
Cell viability was detected by MTT assay. **p < 0.01, *p < 0.05

The cell viability of cardiomyocytes was evaluated using MTT assay. Cell viability was enhanced by 10, 20 and 40 μg/ml Allicin treatment groups(all p < 0.01). Among those groups, cell viability in 20 μg/ml Allicin-treated cardiomyocytes was higher than 10 μg/ml Allicin-treated cardiomyocytes (p < 0.05). However, there is no difference in cell viability between 20 μg/ml and 40 μg/ml Allicin-treated cells. Taken together, 20 μg/ml Allicin treatment was determined to use for further study due to its optimal cardiac protective effect (Fig. 2).

### Allicin alleviated apoptosis induced by HR

**Fig. 3:**
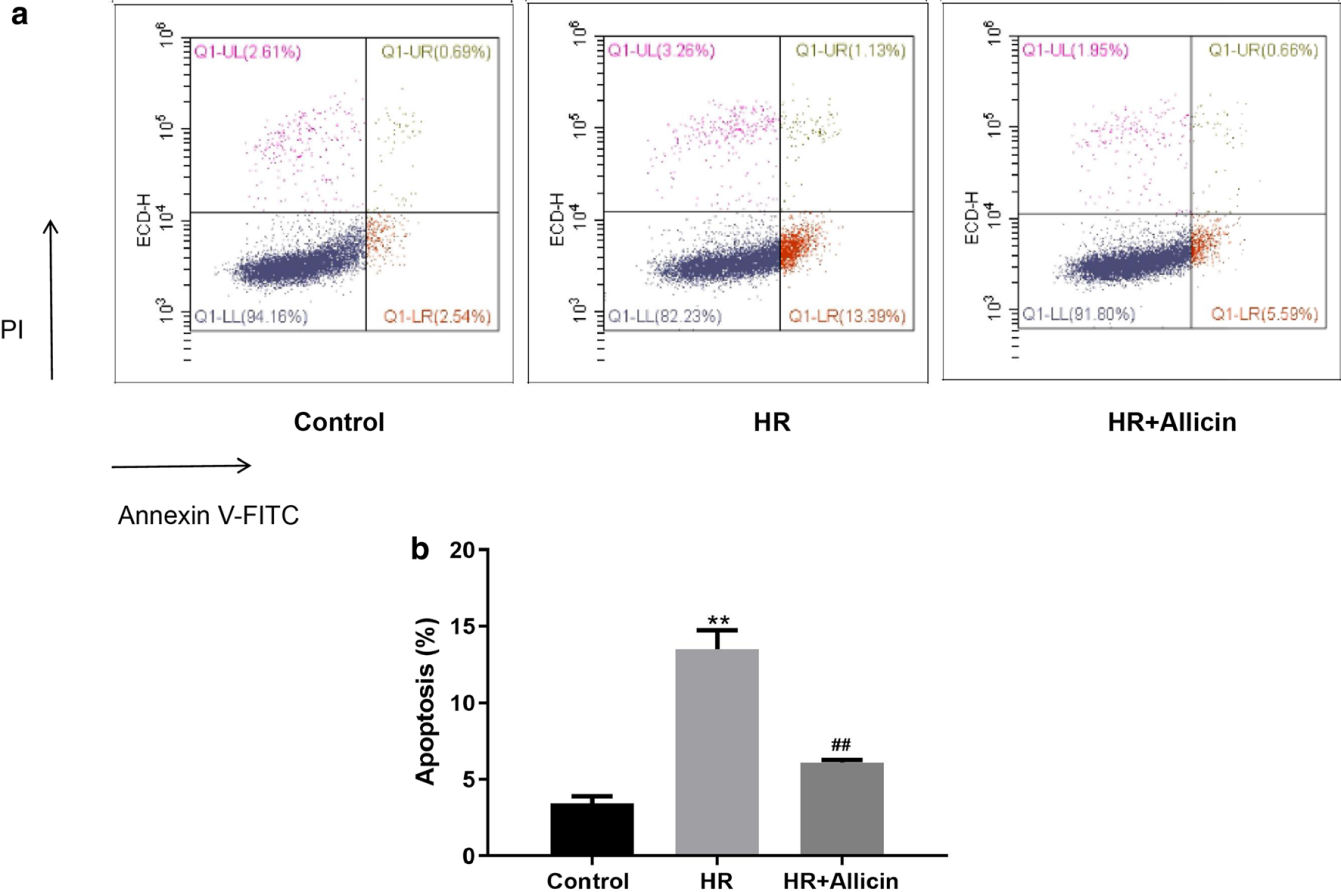
Effects of Allicin on cell apoptosis (Q1-UL: dead cells; Q1-UR: late apoptosis; Q1-LL: normal cells; Q1-LR: early apoptosis). Cell apoptosis was detected by flow cytometry (**a**) and the rate of apoptosis (Q1-UR and Q1-LR) was analyzed (**b**). **p < 0.01 versus the control group; ^##^*p* < 0.01 versus the HR group

Annexin V + / PI- (Q1-LR) cells represent apoptosis in the early stage, while Annexin V + / PI + (Q1-UR) cells represent apoptosis in the late stage. The results showed that HR group was associated with an early apoptosis rate of 12.44 ± 1.04% and late apoptosis rate of 1.07 ± 0.23%. The treatment with Allicin dramatically reduced HR-induced apoptosis, with only 6.11 ± 0.16% cells in the apoptotic phase (Fig. 3).

### Allicin regulated apoptosis-related proteins

**Fig. 4:**
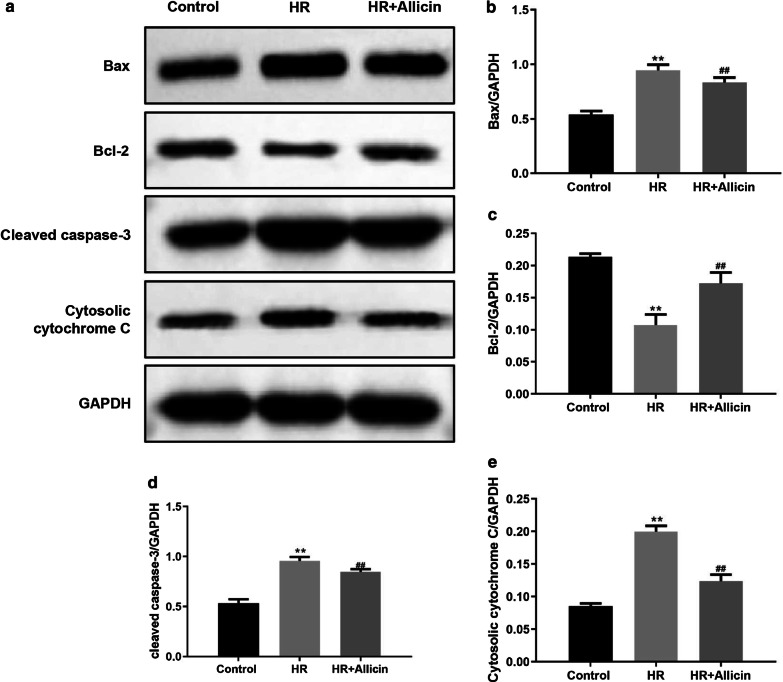
Expression of apoptosis-related proteins by Western blot (**a**). Quantification of the bands associated to the expression of Bax (**b**), Bcl-2 (**c**), Cleaved caspase-3 (**d**) and cytosolic Cytochrome C (**e)** measured by the IPP software. **p < 0.01 versus the control group; ^##^*p* < 0.01 versus the HR group

The results on the expression of apoptosis-related proteins revealed that Allicin induced the expression of anti-apoptotic Bcl-2 protein, and reduced the expression of pro-apoptotic Bax protein, as well as reducing the expression of cleaved caspase-3 and cytosolic cytochrome C(all p < 0.01) (Fig. 4).

### Allicin alleviated the inflammatory cytokines induced by IR

**Fig. 5:**
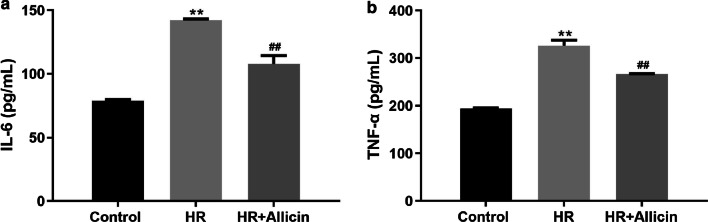
IL-6 (**a**) and TNF-α (**b**) protein expression in each group measured by ELISA. **p < 0.01 versus the control group; ^##^*p* < 0.01 versus the HR group

The expression of TNF-α and IL-6 in the cardiomyocytes in response to HR injury was increased, while Allicin significantly limited their increase (both *p* < 0.01) (Fig. 5).

### Allicin restored the mitochondrial activity

**Fig. 6:**
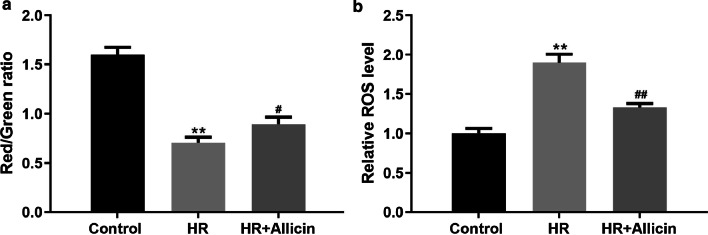
**a** MMP in cardiomyocytes in each group assessed by JC-1 staining. **b** ROS level in each group assessed by DCFDA. **p < 0.01 versus the control group; ^#^*p* < 0.05 versus the HR group. ^##^*p* < 0.01 versus the HR group

The MMP was also measured to further evaluate the potential protective effect of Allicin against HR injury. The MMP was remarkably reduced due to HR injury (*p* < 0.01), because of the compromised mitochondrial activity, but Allicin treatment significantly attenuated the decrease in MMP in cardiomyocytes (*p* < 0.05) (Fig. 6).

In support of this result, the ROS level in cardiomyocytes was also measured, revealing that it was increased because of the HR injury, indicating an ongoing oxidative stress. However, this increase was significantly attenuated when the cardiomyocytes were treated with Allicin (*p* < 0.01) (Fig. 6).

### Allicin restored the expression of PGC1-α, HIF-1α, ET-1, eNOS and TGF-β

**Fig. 7:**
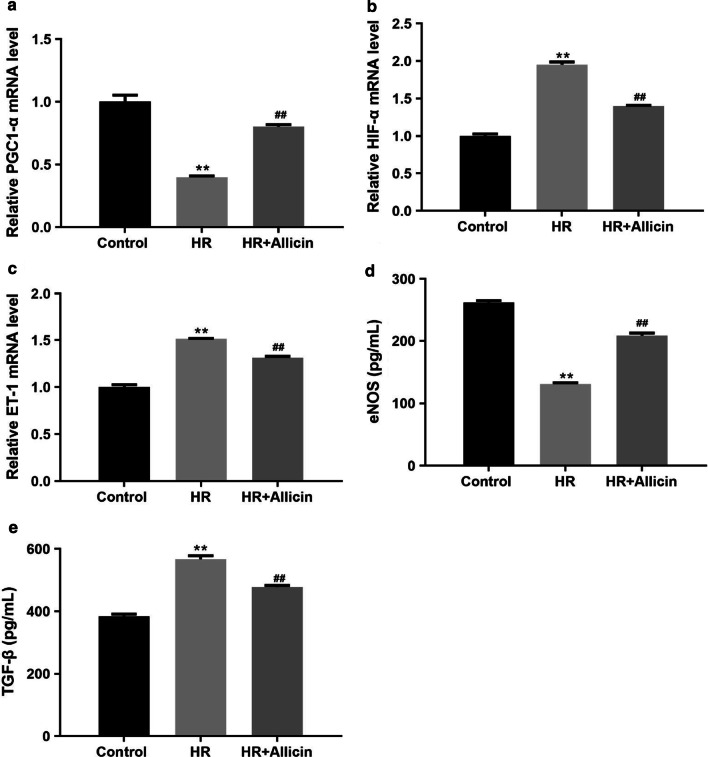
mRNA expression of PGC1-α (**a**), HIF-1α (**b**), ET-1 (**c**) in each group measured by qRT-PCR and protein expression of eNOS (**d**) and TGF-β (**e**) in each group measured by Elisa. **p < 0.01 versus the control group; ^##^*p* < 0.01 versus the HR group

The mRNA expression of Hypoxia-inducible Factor 1-alpha (HIF-1α), endothelin 1 (ET-1) and the protein expression of TGF-β were markedly enhanced in the HR group compared with the control group (*p* < 0.01), but they markedly decreased in the HR + Allicin group (*p* < 0.01), while the mRNA expression of peroxisome proliferator-activated receptor gamma coactivator 1-α (PGC1-α), and the protein expression of eNOS were significantly down-regulated in the HR group (*p* < 0.01), but they were markedly up-regulated in the HR + Allicin group (*p* < 0.01) (Fig. 7).

## Discussion

IR is a major cause of injury in the heart leading to many forms of cardiovascular damage. Although many measures to reduce IR injury are available, currently no standard therapy to prevent the reperfusion injury is found. In this study, we found that Allicin ameliorate cardiomyocytes HR injury in vitro, suggesting that Allicin may have a potential use for the prevention of myocardial IR injury.

Previous studies found that the apoptotic pathway is abnormally activated and excessive apoptosis occurs in IR [9]. This study demonstrated that Allicin treatment significantly inhibited HR-induced apoptosis. Apoptosis is usually accompanied by an increase in the pro-apoptotic protein Bax and a decrease in the anti-apoptotic proteins Bcl-2. The release of cytochrome C from the mitochondria to the cytoplasm is a mechanism involved in one of the pathways of apoptosis, leading to the activation of cleaved caspase-3 that causes cell death by apoptosis [10]. Taken together, our results demonstrated that Allicin effectively alleviated myocardial apoptosis induced by HR.

During IR, damaged cardiomyocytes release endogenous signals that interact with pattern recognition receptors of endothelial cells, mast cells, and cardiomyocytes, leading to the production of pro-inflammatory cytokines and chemokines [11, 12]. Our study demonstrated that Allicin significantly attenuated the increase of IL-6 and TNF-α in cardiomyocytes caused by HR, thus alleviating the inflammation of the heart.

Then the effect of Allicin on mitochondrial function was investigated. During normal perfusion, mitochondria consume oxygen and produce adenosine triphosphate (ATP), which functions in the maintenance of a dynamic ROS balance. However, intracellular and mitochondrial effects caused by IR, such as Ca2 + overload, inadequate ATP resynthesis and the burst of ROS, cause the opening of mitochondrial permeability transition pore, leading to the collapse of MMP that cause apoptosis [13]. According to our study, Allicin alleviated both ROS production and the reduction in MMP, thus protecting the mitochondrial function to some extent.

Besides, Allicin treatment significantly restore the expression of eNOS and PGC1-α, and significantly decreased the expression of ET-1, HIF-1α and TGF-β. Current studies show that eNOS/PKG pathway is protective against IR injury [14], suggesting a potential mechanism used by Allicin to protect cardiomyocytes. PGC1-α is a transcriptional activator playing an important role in regulating mitochondrial biogenesis and myocardial metabolism. A previous study showed that PGC1-α up-regulates mitochondrial antioxidants [15]. ET-1 is one of the most effective endogenous vascular constrictors that mediate a variety of reactions, including endothelial dysfunction, vascular contraction, leukocyte activation, and cell proliferation [16]. High levels of ET-1 impair the production of endothelial nitric oxide. Some studies showed that IR injury leads to the release of ET-1, which stimulates calcium overload and apoptosis, and ET-1 inhibitors is able to reduce IR injury [17]. HIF-1α is a transcription factor that induces cell hypoxia. A low oxygen environment increases the expression of HIF-1α mRNA. Although many studies show that HIF-1α exerts a protective effect on myocardial IR, other studies suggest that HIF-1α exerts a cardio-deleterious effect leading to apoptosis [18, 19]. Our study found that Allicin slightly reduced HIF-1α expression caused by HR, consequently protecting the cardiomyocytes, thus representing a potential mechanism to attenuate apoptosis caused by IR. Although the role of TGF-β is still controversial, previous studies showed that the downregulation of TGF-β expression reduces myocardial IR injury [20]. However, many cytokines and substances affect the transcription of these genes, meaning that further studies are needed to confirm the specific molecular mechanism exerting a cardio-protection found in our study. Altogether, these data suggest that Allicin may be a new therapeutic option for myocardial IR (Additional file 1).

## Conclusions

Despite our results need to be confirmed by additional studies, they suggested a protective effect of Allicin on cardiomyocytes against HR damage by reducing apoptosis, inflammation and mitochondrial injury, thus providing a basis for its potential use in the treatment of myocardial IR.

## Supplementary Information

**Additional file 1.** Original Western blot data.

## Data Availability

The data sets used and/or analyzed during the current study are available from the corresponding author on reasonable request.

## Abbreviations

IR: Ischemia–reperfusion
Bax: Bcl-2 Associated X Apoptosis Regulator
Bcl-2: B-cell lymphoma 2
IL-6: Interleukin-6
TNF-α: Tumor necrosis factor-α
TGF-β: Transforming growth factor-β
eNOS: Endothelial nitric oxide synthase
ROS: Reactive oxygen species
HIF-1α: Hypoxia-inducible Factor 1-alpha
ET-1: Endothelin 1
PGC1-α: Peroxisome proliferator-activated receptor gamma coactivator 1-α
ATP: Adenosine triphosphate
