# Supplementary material for: Allicin attenuates myocardial apoptosis, inflammation and mitochondrial injury during hypoxia-reoxygenation: an in vitro study
Source: BMC Cardiovasc Disord. 2021 Apr 21;21:200. doi: 10.1186/s12872-021-01918-6 (PMC8059159; doi:10.1186/s12872-021-01918-6)
Supplement: Supplementary file 1 — Additional file 1. Original Western blot data. [file 12872_2021_1918_MOESM1_ESM.pptx]

## Slide 1
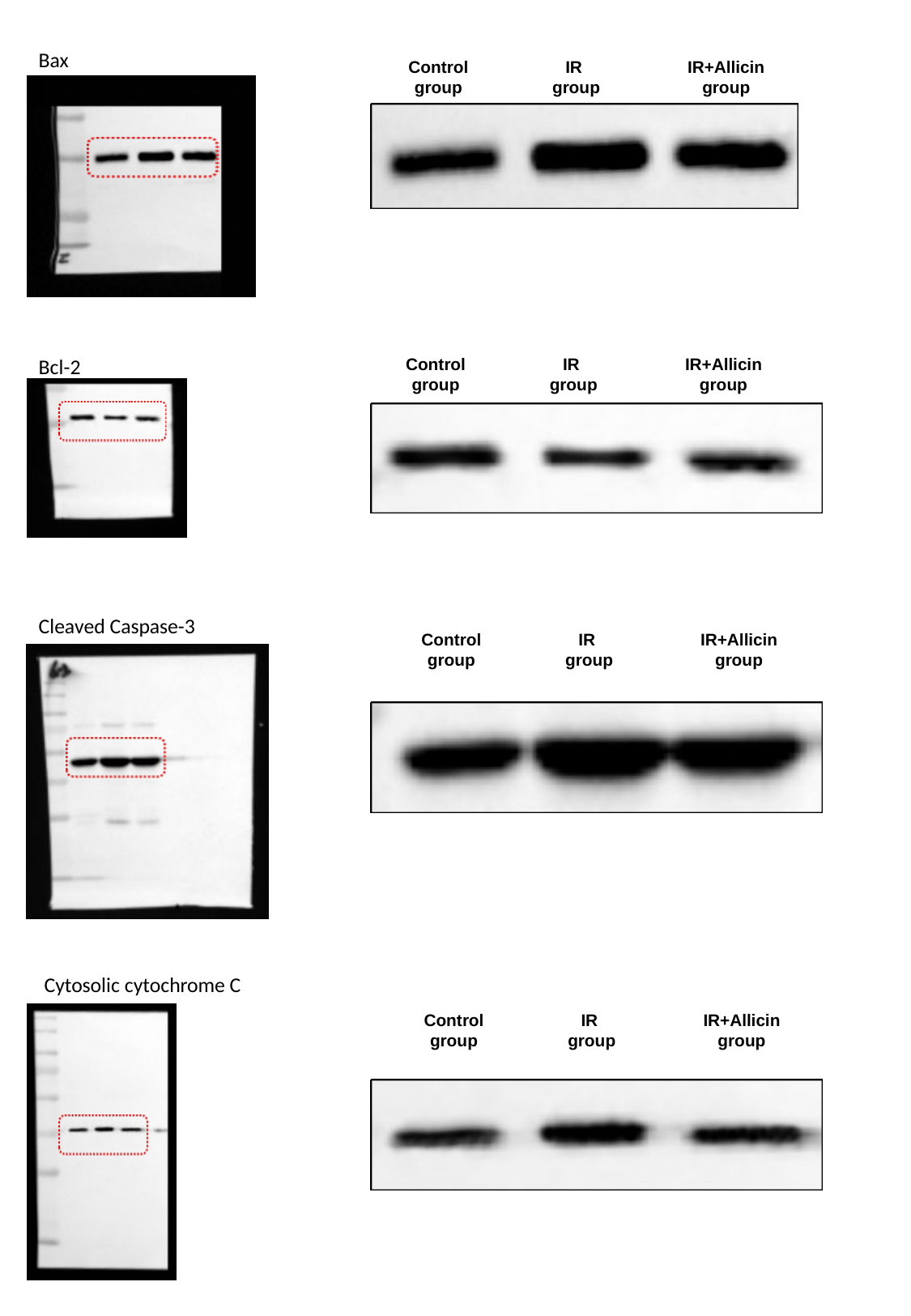

Bax
Control group
IR
group
IR+Allicin
group
Bcl-2
Control group
IR
group
IR+Allicin
group
Cleaved Caspase-3
Control group
IR
group
IR+Allicin
group
Cytosolic cytochrome C
Control group
IR
group
IR+Allicin
group

## Slide 2
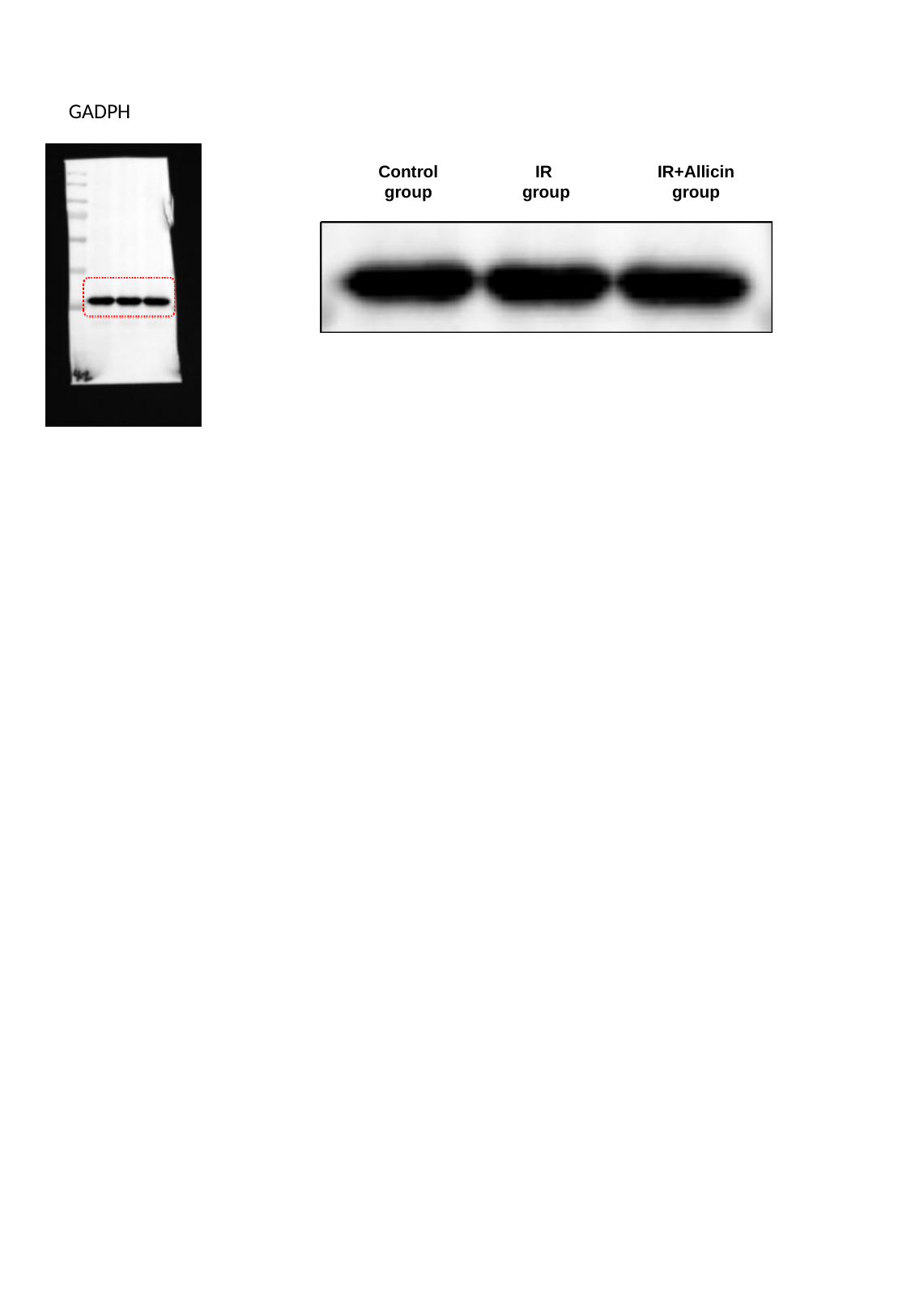

GADPH
Control group
IR
group
IR+Allicin
group
